# The Role of the Environment in Eliciting Phantom-Like Sensations in Non-Amputees

**DOI:** 10.3389/fpsyg.2012.00600

**Published:** 2013-01-18

**Authors:** Elizabeth Lewis, Donna M. Lloyd, Martin J. Farrell

**Affiliations:** ^1^School of Psychological Sciences, The University of ManchesterManchester, UK

**Keywords:** amputation, embodied experience, interpretative phenomenological analysis, peripersonal space, phantom limb, proprioception, rubber hand illusion

## Abstract

Following the amputation of a limb, many amputees report that they can still vividly perceive its presence despite conscious knowledge that it is not physically there. However, our ability to probe the mental representation of this experience is limited by the intractable and often distressing pain associated with amputation. Here, we present a method for eliciting phantom-like experiences in non-amputees using a variation of the rubber hand illusion in which a finger has been removed from the rubber hand. An interpretative phenomenological analysis revealed that the structure of this experience shares a wide range of sensory attributes with subjective reports of phantom limb experience. For example, when the space where the ring finger should have been on the rubber hand was stroked, 93% of participants (i.e., 28/30) reported the vivid presence of a finger that they could not see and a total of 57% (16/28) of participants who felt that the finger was present reported one or more additional sensory qualities such as tingling or numbness (25%; 7/28) and alteration in the perceived size of the finger (50%; 14/28). These experiences indicate the adaptability of body experience and share some characteristics of the way that phantom limbs are described. Participants attributed changes to the shape and size of their “missing” finger to the way in which the experimenter mimed stroking in the area occupied by the missing finger. This alteration of body perception is similar to the phenomenon of telescoping experienced by people with phantom limbs and suggests that our sense of embodiment not only depends on internal body representations but on perceptual information coming from peripersonal space.

## Introduction

The sense of one’s own body is largely determined by the multisensory integration of visual, tactile, proprioceptive, and kinesthetic (and possibly auditory) information. Multisensory information about the body comes not only from the body itself, but also from the surrounding environment with which the body interacts. Though we are aware of the resulting sense of embodiment, we are not normally aware of the multisensory integration that produces it. This is only revealed in abnormal situations, such as body illusions, of which the rubber hand illusion (RHI; Botvinick and Cohen, 1998) is a striking example, and pathological phenomena, such as the experience of phantom limbs. Given that the RHI and phantom limbs both result from the alteration of the sensory input that creates the sense of embodiment, the goal of the present paper was, firstly, to determine whether an analog of the phantom limb experience could be created in participants without an amputation through a variation of the RHI. Secondly, we wanted to demonstrate the usefulness of first-person methodology in investigating the experience of embodiment. Although embodiment has been the subject of a wealth studies investigating normal body representation (e.g., Botvinick and Cohen, 1998; Armel and Ramachandran, 2003), only a handful of studies have sought to understand the phenomenological aspects and determinants of subjective experience underlying abnormal body representations, such as phantom limbs. Such investigations are rare because they are difficult to carry out: the phantom limb is often a transitory phenomenon and, even when it is relatively long-lasting, the experience of pain that is associated with it makes it difficult for patients to reflect analytically on their experience of the phantom limb. The ability to create an analog of the phantom limb experience in intact participants could, therefore, prove to be an important tool in investigating the sense of embodiment and how it relies upon our interaction with the environment.

Our sense of embodiment depends on both bottom-up factors, in the form of incoming sensory information, and on top-down factors, such as the body schema. The body schema acts as a constraint on the sensory processes that underpin body representation by forming a tacit expectation of the body’s possible movements (Head and Holmes, 1911; Cardinali et al., 2009). Previous studies have shown that when the expectation of the body schema is violated (as in the case of paralyzed limbs) this may, in some cases, lead to discomfort and a sense of “disownership” of the limb (Moseley et al., 2008) so that the person no longer feels that the limb belongs to them. Embodiment, therefore, can be seen as dependent on the functional capabilities of the body: if the body part is no longer in use or useful then it may become “disowned.” This holistic sense of self arises through the interaction between the representation of the body modified though multisensory integration and the perception of being in control of the body (i.e., the sense of agency), which forms judgments of self-attribution and limb ownership.

However, this holistic embodiment breaks down when alterations in body representation occur through neural damage or through a perceptual illusion. For example, in the RHI, a viewed prosthetic hand is stroked in precise spatial and temporal synchrony with the stroking of a participant’s concealed hand. The majority of people report perceiving the touch from the rubber hand as if it were part of their own body. In other words, a “sense” of embodiment is transferred to an external object and the real hand is disowned. The subjective experience of this feeling of ownership over the rubber hand has generally been measured using self-report questionnaires (e.g., Botvinick and Cohen, 1998; Armel and Ramachandran, 2003; Ehrsson et al., 2004, 2005, 2008; Mussap and Salton, 2006; Schaefer et al., 2006; Durgin et al., 2007; Kitadono and Humphreys, 2007; Lloyd, 2007; Tsakiris et al., 2007; Haans et al., 2008; Longo et al., 2008; Moseley et al., 2008; Capelari et al., 2009; Dummer et al., 2009; Kammers et al., 2009; Schütz-Bosbach et al., 2009; Shimada et al., 2009). Several authors have argued that the feeling of ownership over the rubber hand is induced because vision and touch capture converging, correlated information and this forms a meaningful percept, i.e., the visual perception of a hand being touched co-occurs with the tactile sensation of the hand being touched. This perception becomes dominant, and the conflicting proprioceptive information, which indicates the true position of the participant’s hidden hand, is adapted leading to proprioceptive distortion (measured as the distance the intact hand is believed to have moved from its original starting point toward the rubber hand). Human functional neuroimaging studies have revealed that visual, tactile, and proprioceptive inputs relating to limb position are integrated in the premotor and parietal cortices (Lloyd et al., 2002) and the degree of premotor activation shows a linear relationship to how participants subjectively rate the illusion (Ehrsson et al., 2004, 2005).

The RHI demonstrates that the sense of embodiment is strongly influenced by the sensory information produced through interaction with the environment. In the case of the RHI, it is not only proprioceptive information generated internally by the body, but also visual and tactile information generated through interaction with the experimenter, that results in an altered sense of embodiment. Indeed, as we have seen, the proprioceptive sense is actually distorted so that it fits to a greater extent with the visual and tactile information. The idea that the relationship between the body and the world is integral to the overall sense of embodiment (i.e., we get a sense of our own bodies via their interaction with the world) also helps to make sense of other phenomena. In asomatognosia, for example, in which patients have no sense of ownership over one of their limbs, placing the neglected limb into the attended (i.e., contralateral) body space restores multisensory processing (Moro et al., 2004). In complex regional pain syndrome (CRPS), not only does the affected limb feel cooler but that whole side of space is physiologically dysregulated as when the affected limb was moved to the opposite side of space it got warmer (similarly when the “good hand” moved to the affected side of space it got cooler; Moseley et al., 2012). These clinical findings point to the same conclusion as experimental studies of the RHI: our sense of embodiment is dynamic and dependent on the body’s positioning within the space that surrounds it.

Peripersonal space is the region surrounding the body that acts as the interface between the body and the environment for defensive and purposeful action (Cardinali et al., 2009). Neurophysiologists have defined peripersonal space based on the spatial limits of visual receptive fields of individual neurons most often found in the parietal and premotor cortices of non-human primates (e.g., Graziano et al., 1997). For example, in monkey posterior parietal cortex, peripersonal space encoding involves ventral intraparietal area, which contains visuotactile bimodal cells for the face, arm, and hand. These cells use a body-part-centered reference frame to represent visual space around the body, such that the visual receptive field of the bimodal cell is bound to the space surrounding the tactile receptive field of a particular body part. Visual signals from a region of the body can therefore activate a somatotopic map relating to that body part and can remap with changes in posture (Graziano and Gross, 1994). Recent evidence from a functional magnetic resonance imaging (fMRI) study suggests that intraparietal areas similarly encode objects in the peripersonal hand space of humans and may also have a role in visually guided grasping (Makin et al., 2007). Thus, in addition to spatial location influencing our sense of embodiment, the body plays a role in structuring peripersonal space (i.e., it is body-part-centered). There are, in other words, reciprocal relations of influence between the sense of our bodies and our perception of peripersonal space.

One of the ways in which we can get a handle on the sense of embodiment and how it arises is through the investigation of abnormal embodiment. In cases such as these, the normally “invisible” processes that underlie embodiment become more apparent than is usually the case. One of the most striking forms of abnormal embodiment is the phantom limb. Ambroise Paré first reported phantom phenomena in amputee soldiers in the mid sixteenth century. However, it wasn’t until Silas Weir Mitchell published the first detailed study (where the term “phantom” was used) in the nineteenth century that phantom limb phenomena (PLPh) became recognized as real sensory experiences and not psychiatric symptoms (Mitchell, 1871). PLPh have been defined as a “continuous awareness of a (or part of a) non-existing or de-afferented body part with specific form, weight, or range of motion” (Ribbers et al., 1989) and have been reported not only in amputees, but also in paraplegics and people with a congenital absence of limbs (Melzack and Loeser, 1978). PLPh may be felt as pins and needles, itching, tingling, or numbness (Katz, 1992; Montoya et al., 1997) whereas others experience embodiment without sensation (i.e., they know that the phantom is there but have no feeling in it – Hunter et al., 2003; Richardson et al., 2006). Movement and position sense may also remain intact in the phantom limb and can be spontaneous (i.e., spasm) or volitional. For example, some patients report gesturing during conversations and can carry out finger-aided counting (Ramachandran, 1993; Saadah and Melzack, 1994; Ramachandran and Hirstein, 1998; Brugger et al., 2000). Some patients also report the sensation of telescoping, in which the phantom shortens over time until only the digits remain on the end of the stump (Weiss and Fishman, 1963; Jensen et al., 1984). There may also be super-added sensations of feeling a watch on the arm or clothing against the phantom skin (Wesolowski and Lema, 1993) indicating that memory systems may help to maintain the phantom experience (Katz and Melzack, 1990; Richardson et al., 2006).

Capturing the subjective experience of embodiment, whether that be the experience of phantom limbs or the RHI, tends to rely on self-report questionnaires using Likert scales in which participants rate their level of agreement or disagreement with statements describing the experience (e.g., “the rubber hand feels like my hand”). But these questionnaires, which use a limited range of items, may have obscured the important subcomponents of embodied experience. A more rigorous analysis of first-person accounts can provide a scientific description of the phenomena and serve as the basis for quantification. An excellent example of this is a study of the alien hand experiment (TAHE) by Sørensen (2005); first reported by Nielsen (1963). In TAHE, the participant is asked to draw a series of objects, which he/she can only see via a tilted mirror. On some trials, the mirror is tilted in such a way that the participant actually sees the experimenter draw the object instead, giving the subjective experience that the participant is not in control of their body. By using first-person reports of the phenomena as experienced by the participant, categories for quantification were disclosed by the data themselves and it was possible to show that concepts such as agency and body schema do reflect real phenomenological aspects of experience.

A more recent study confirmed the utility of first-person methods for the study of embodiment. First-person accounts of subjective experience during the RHI were analyzed using interpretative phenomenological analysis (IPA; Lewis and Lloyd, 2010). IPA has its roots in symbolic interactionism; as such, it is concerned with how meanings are constructed by individuals both on a social and personal basis (Smith and Osborne, 2007). People may experience the same objective event but the meanings that each person attaches to it may be very different, and these personal meanings and feelings will be reflected in the language used to describe the experiences. In IPA, the researcher attempts to find the themes and categories that emerge naturally from the freely produced discourse of participants rather than imposing a preconceived set of categories on participants’ responses. Such an approach is advantageous for investigations of novel phenomena, such as the RHI and PLPh, which may never have been experienced before, and therefore require an open and flexible approach to the language used by participants. In the study by Lewis and Lloyd (2010) IPA revealed four main themes of embodied experience during the RHI: recalibration of the body schema; violation of the body schema; multisensory integration; and illusory experience over time. Furthermore, the report of agency was a significant predictor of the amount (in centimeters) of proprioceptive distortion. This study shows how first-person methodologies can be empirically rigorous and how the introspective interview provides a rich, detailed account of embodied experience.

The aim of the present study was to establish, through the use of first-person phenomenological methods, whether an analog of the subjective experience of a phantom limb could be induced in intact participants by having them take part in a modified version of the RHI. We manipulated the RHI paradigm by removing the ring finger from a right rubber hand. Through this manipulation we aimed to discover whether participants, after being induced to feel ownership over the rubber hand, would feel the presence of the absent finger in a way analogous to the feeling of a phantom limb.

## Materials and Methods

### Participants

Thirty right-handed participants (13 males, 17 females; aged 19–29 years, mean age of 22.5 years) were recruited from the University of Manchester via opportunity sampling. The study was approved by the School of Psychological Sciences research ethics committee. Participants were screened for tactile and proprioceptive impairments of the right hand. They received five pounds compensation for their time.

### Materials

**Figure 1:**
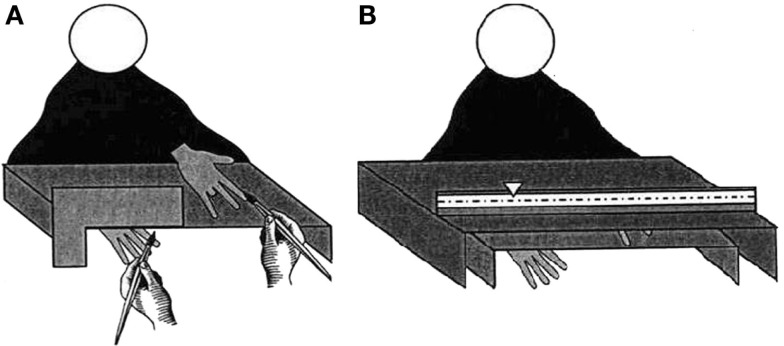
**(A)** Participants viewed a rubber hand with a missing finger on top of an open-sided box while their real hand was hidden from view beneath the box. The experimenter stroked both the real and the rubber hand simultaneously through one of the box’s open sides; **(B)** A larger box with a ruler was used to measure participants’ estimates of the position of their real hand on the basis of proprioception alone. This was done before and after inducement of the RHI and only the experimenter, seated opposite the participant, could see the markings on the ruler.

Participants sat at a table across from the experimenter and placed their right hand inside a specially constructed box (40 cm × 27 cm × 7 cm; Figure 1A). The ring finger of their right hand was placed on a predetermined spot concealed from view. The side of the box facing the experimenter was open so that the experimenter could stroke the participant’s hand. A prosthetic (right) rubber hand was placed palm down on top of the box so that there was a 10 cm horizontal separation between the rubber hand and the participant’s real hand. The rubber hand used in the standard RHI condition was intact and the rubber hand used in the missing finger condition was identical except that the ring finger had been cut off prior to the experiment.

The rubber hand was placed in full view of the participant and covered from the wrist by a fake sleeve so that it was a plausible extension of the participant’s right arm. A second larger box with a hidden ruler (46 cm × 27 cm × 12 cm; Figure 1B) was used to conceal the equipment and take proprioceptive measurements. Both boxes were covered with felt to conceal any distinguishing marks which may have aided localization.

### Procedure

Participants took part in both the standard RHI and the missing finger versions. Participants took part in the intact version of the illusion first as it was necessary to establish the basic illusion before the missing finger variation was performed. As it was the missing finger version that was the focus of interest, this order of presentation had the additional benefit of getting participants used to talking about the illusion. Participants had a 2–3 min break between the illusions where they were allowed to move freely. In both conditions, participants placed their right hand inside the apparatus while their left hand rested comfortably on the table beside the apparatus. Participants were informed that they would take part in an illusion and that they should describe their experience of this illusion as fully as possible. In addition, they were asked to watch the rubber hand and keep their right hand still. A single initial proprioceptive measure was obtained by placing the larger box over the equipment and sliding a marker across the hidden ruler until the participant indicated that it was over the location of their ring finger. Then the audio recorder was started. In both conditions the fingers of the rubber hand (excluding the ring finger) were stroked simultaneously with the participant’s own fingers. If the participants did not provide a description of their experience after 1.5 min they were asked to describe how they felt about the rubber hand while they watched it being stroked. The RHI was considered established when the participant stated that either “the rubber hand felt like their hand,” “the touch was felt in the rubber hand,” or they felt that they could “move the rubber hand volitionally.” Then the experimenter stroked the ring fingers simultaneously. During the missing finger condition, the experimenter mimed the stroking of a finger in the empty space that would have been occupied by the ring finger of the rubber hand while simultaneously stroking the participant’s ring finger. Participants were reminded to describe their experience of the illusion. To encourage this they were prompted with the following questions: how does this feel? How do you feel about your real hand? Is this a comfortable experience? The questions were purposefully vague and their order was not fixed. After 3 min, a second proprioceptive measure was taken in the same manner as the first.

### Analysis

Audio recordings were transcribed verbatim and analyzed using Interpretative Phenomenological Analysis (IPA: Smith, 1996; Smith et al., 1999).

**Figure 2:**
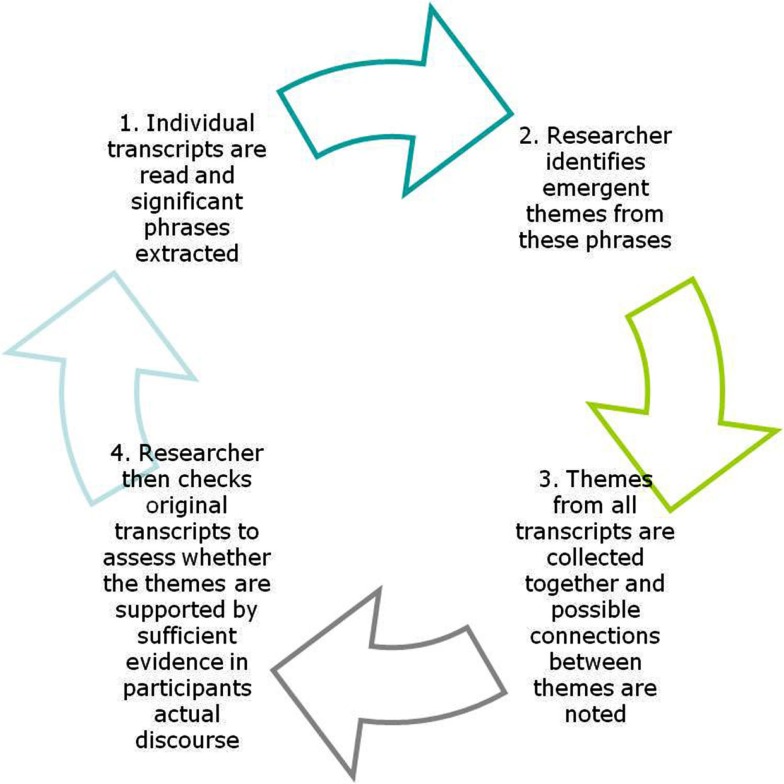
**The processes involved in Interpretative Phenomenological Analysis**. The researcher will go through as many iterations of this cycle as he/she feels is necessary to capture adequately the themes and sub-themes that emerge from participants’ discourse.

Transcripts were analyzed individually using a two stage process (Figure 2 illustrates the elements of this analysis). After familiarization with a transcript, the left margin was used to code the themes of the adjoining parts of the transcript. Then, similar or related codes were collapsed into broader themes, which were noted in the margin on the right. Once this was complete for every transcript the data were considered as a whole. Variations in themes across transcripts were used to establish broader themes which could demonstrate the structure of the experience across the entire sample. Specifically, the themes highlighted differences between the transcripts in each condition. Themes are presented with examples of the participants’ discourse as evidence as well as the identifying number for that participant in parentheses after each example of discourse. Quantitative measures of proprioceptive distortion were analyzed using analysis of variance (ANOVA) and SPSS v.16.

## Results

### Quantitative results

**Figure 3:**
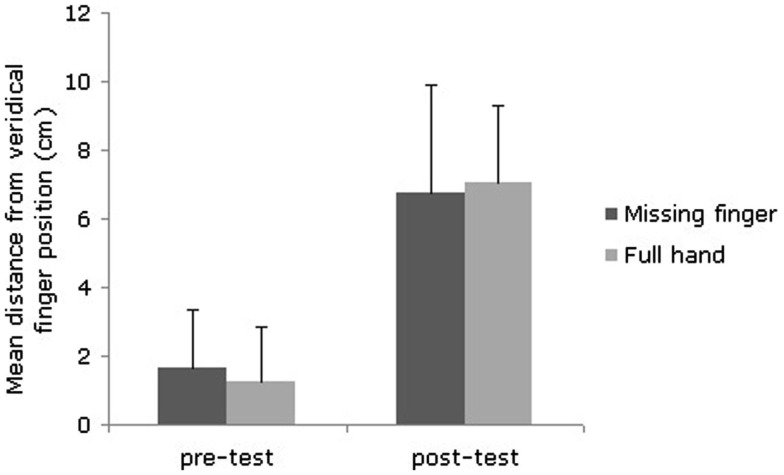
**The difference (in cm) between participants’ proprioceptive estimate of their hidden finger’s position before and after induction of each version of the RHI**.

A 2 × 2 repeated measures ANOVA (before/after illusion × finger-absent RHI/finger-present RHI) was conducted to assess whether proprioceptive judgments of the position of the ring finger of the right hand were shifted away from its objective position before and after each illusion (finger-present vs. finger-absent). There was a significant main effect of the time point of the proprioceptive measure [*F*(1, 29) = 221.34, *p* < 0.001], but there was no main effect of finger presence (*F* < 1) or an interaction between the time point and finger presence [*F*(1, 29) = 1.41, *p* = 0.245]. Participants judged their finger to be significantly further away from its actual location after the illusion (present *M* = 7.03 cm, SD 2.27 cm vs. absent *M* = 6.80 cm, SD 3.13 cm) than they did at baseline (present *M* = 1.23 cm, SD 1.61 cm vs. absent *M* = 1.63 cm, SD 1.71 cm) but the presence or absence of the finger did not influence the proprioceptive judgments (See Figure 3).

### Qualitative results

#### Theme 1: “my invisible finger” – an altered body form changes the effect of multisensory integration on somatic experience

When the intact fingers of the rubber hand were stroked, the participants’ descriptions were the same in both conditions and their comments reflected the experience associated with the standard RHI. They described how the rubber hand felt as though it was their hand or the touch sensations felt located in the rubber hand, whilst their awareness of their own hand had diminished. During the missing finger condition, participants stated that they could still perceive their ring finger even though it was missing on the rubber hand. Many participants believed that this was the aim of the experiment and emphasized that they could be fooled into owning another hand but they could not be fooled into perceiving their finger as absent. So just seeing and feeling ownership over an altered body form – in this case the rubber hand with a missing finger – does not induce changes in the phenomenal experience of body form; the hand is still experienced as intact:

“It feels like this (rubber) hand is my hand again…like my hand is up there. But it doesn’t feel like I’m missing a finger, it doesn’t feel like my finger disappears or anything. It still feels the same as normal.” (4)

When the missing finger area was stroked simultaneously with the participants’ ring finger, 93% of participants (i.e., 28/30) reported that they could perceive their ring finger extending out from the stump on the rubber hand even though they could see that it was not there. They discounted the visual information indicating that the finger was missing and the correspondence of tactile cues and visual information from the mimed stroking was sufficient to elicit a percept of their ring finger. The finger was predominantly described as “invisible” or using a metaphor to convey a physical entity which cannot be seen, for example, “my finger is made of glass” or “painted to match the color of the box.” Two of the participants described an alternative experience of the illusion: when the area of the missing finger was stroked they instantly reported a holistic shift in awareness to their hidden hand. The following quotes demonstrate these two contrasting ways of describing the illusion

“Oh my god, I just felt like my hand was invisible! The finger isn’t there but I feel like it should be so I feel like it is there, I just can’t see it. The rubber hand before felt like it was my hand and this also feels like it is my hand. I just feel like Harry Potter’s invisibility cloak has been draped over my ring finger. It is there I just can’t see it. I feel like if I moved my finger toward it I would be able to touch it even though it is not there. Even though I can’t see it, it doesn’t feel like its missing or not there.” (2)

“I was getting the feeling that this rubber hand was my real hand but as soon as I can see that I don’t have a finger here, I can get the feeling of my (actual) hand, it suddenly changes…You get your normal state of mind back when you can see that you don’t have a finger there even when you can get the feeling there.” (29)

The majority of participants described their RHI experience as being akin to their normal somatic experience and they remarked that they could “actually feel the finger.” But 25% of the participants (7/28) also reported a reduction in sensation or an increase in somatic intensity, pins and needles, or numbness when they could perceive an invisible finger:

“It feels a bit tingly but it feels like you are still carrying on (stroking) down the finger. I don’t know if, because I’m watching it, I tense up, but it feels more strained or tingly.” (4)

“I don’t feel the sensations as much as before. I definitely feel a loss of sensation at that point (the stump). My finger feels numb.” (9)

#### Theme 2: the dynamic relationship between the environment and the mental representation of the body is altered in the missing finger illusion

**Figure 4:**
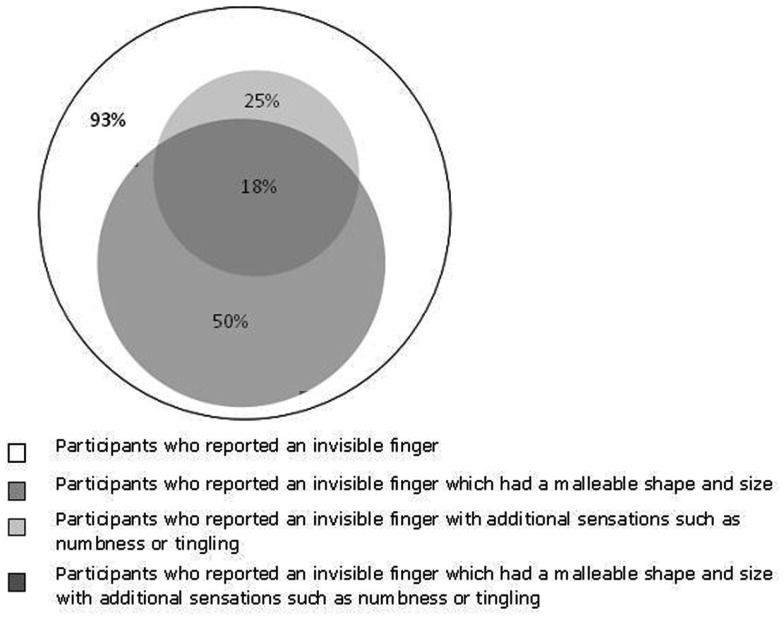
**A Venn illustrating the proportion of participants who described their feeling of the missing finger in various ways**. A definition of each characteristic emerged from the qualitative analysis and was subsequently used to quantify the proportion of participants who reported each characteristic. The number of participants who reported each adjective is presented below in brackets. The white circle represents the 28 participants who described their ring finger as invisible (20), or used another metaphor to describe something with a physical presence without a visual image, e.g., a camouflaged finger (8). The dark gray circle represents the 14 participants who reported an invisible finger which had a malleable shape and size. These participants reported the following adjectives when comparing their invisible finger percept to normal embodiment or their experience during the RHI: longer (7), extending (2), stretching (2), flatter (2), bumpier (1), squashier (1), swollen (1), clenched fingers (1). The light gray circle represents the seven participants who reported additional sensations in their invisible finger. These participants reported the following adjectives when comparing their invisible finger percept to normal embodiment or their experience during the RHI: tingly (2), more intense (4), numb (3), tense (2), aching (1), cold (1), heavy (1). The region of gray overlap represents the five participants who reported an invisible finger which was both malleable in form and had additional sensations.

During the RHI, the participants’ sense of embodiment is changed by manipulating the sensory input generated by interaction with the environment. In the missing finger illusion the seen location of mimed finger stroking can determine the perceived “physical” qualities of the missing finger. It was difficult to trace the outline of the finger perfectly and slight, unintentional variations in the experimenter’s precise stroking action naturally arose in the course of individual testing sessions. These variations influenced the perceived shape of the invisible finger. When the experimenter’s stroking finger was seen to deviate from the expected shape of a finger, the participants reported that their invisible finger had changed shape, for example, by becoming longer or flatter etc. This was spontaneously reported by 50% of the participants (14/28) and the number of participants that reported different types of alteration in finger shape is given in Figure 4.

The following quote is illustrative of some participants’ spontaneous reaction to variations in the mimed stroking of the missing finger:

“Arrgh! That totally felt out in space. That was amazing. It’s just so compelling…but it feels like the invisible finger is kind of less solid than my other fingers, it’s kind of a bit squashier. Cos I’m looking very closely, I can see it’s very hard for you to trace a finger realistically, but I’m kind of adopting that, so my invisible finger isn’t as solid as the other fingers around it.” (6)

The changes in form could be quite large, for example, the finger could be perceptually extended by 3 cm to the edge of the experimental equipment. But the experiences reported were not limited to perceived shape differences. For example, some participants attributed the changes to their own active movements even though they were not moving. In addition, changes to the shape of the invisible finger were, in two participants, also associated with changes to perception of the rest of the hand. The seen location of interaction, therefore, sometimes had a holistic effect on the whole hand representation and not just on the invisible finger:

“There I felt like my finger was reaching out quite a long way. I’ve basically got a big long ghost finger and it goes up to the boundary of wherever you make it go. That felt like my finger was really extended out, like the other fingers are clenched in a bit. I felt like I was pushing that (ring) finger out really far, like that one was extended and the others were kind of flexed.” (6)

Perceived changes in the form of the hand were not reported during the standard RHI. When comparing the two conditions, the standard RHI was described as “normal” or “not unusual.” There was a range of reactions to the missing finger condition. No participants reported a painful or uncomfortable experience but some did experience it as aversive. The participants were aware that their experiences were illusory but this did not diminish their impact. It was possible to experience an invisible finger but the participants described it as being accompanied by a sense of wrongness or as somehow being invalid. They were motivated to resolve the conflict between the visible absence of the finger and the felt presence of touch through active movement:

“When you stroked up to the stump, it felt about right and then I was almost expecting not to feel anything (past the stump), as ridiculous as that sounds. And when I still felt it, it was like an extreme sense of wrongness to be honest. Almost like I immediately wanted to move and shake my hand. The urge to move is quite marked. I’d say it was frustrating in that my immediate reaction would be to clench and unclench my fist to make sure everything is working.” (11)

In fact, two participants not only felt this urge to move their fingers but actually removed their hand from the equipment.

During the standard RHI, participants often report that they feel as though their hand has taken on the rubbery texture of the rubber hand. Cross modal texture effects were also reported in the missing finger condition; however, these related to textures within the environment as opposed to the surface texture of the body. Two participants reported that they could feel the felt covered surface of the box on which the rubber hand was resting even though their hand was resting on the smooth surface of the table:

“When you were pressing down, I actually felt (that) it should hurt more than that because you went so low into the table; like my invisible finger was being pressed but then it wasn’t. It’s very realistic. When you press on the fingernail at the end…that very much feels like I’m pressing into the felty surface. So now I can really feel it on the felty stuff.” (6)

## Discussion

In both versions of the RHI, finger-present and finger-absent, errors in the proprioceptive judgments of finger position were larger (i.e., closer to the position of the rubber hand) after the induction of the illusion than they were prior to the induction of the illusion. There was no significant difference in judgments about finger location between the two forms of the illusion, and so both elicited a similar amount of proprioceptive adaptation. Even when there was no rubber finger to embody in the missing finger condition, the participants felt their real finger to be located in the area where the missing finger would have been. First-person reports confirmed that participants felt their finger protruding from the stump of the rubber hand and that they could feel an “invisible finger” in the location where the missing finger would have been. Although perceived finger location was comparable in each version of the illusion, the subjective experience of finger presence was different in the two cases. In the missing finger condition, participants typically reported somatic experiences such as numbness, which attenuated the strength of the reported tactile sensations. In addition, in the missing finger condition the perceived form of the invisible finger was not fixed and at certain times it was reported to change size and shape. These perceptions were often accompanied by a sense of “wrongness” and a recognition that these sensations should not happen. This was not the case in the standard, finger-present, version of the RHI, in which normal feelings of body awareness are experienced as located in the rubber hand.

In the current study 93% of the participants reported that they felt their finger extending out from the stump of the rubber hand and this is comparable to the incidence of a continued experience of a removed body part in amputees, which is as high as 98% in some samples (Ramachandran and Hirstein, 1998). Within the general phantom limb experience, however, there is a range of more specific phenomena that are only seen among subgroups of amputees. Some, for example, can describe the shape, size, and range of movement of the phantom (Katz, 1992; Richardson et al., 2006), whereas others only have a more vague sense of the phantom’s presence. Some, in addition to the sense of the phantom’s presence, have other sensations, such as pain or tingling. The prevalence of these more specific types of experience has been difficult to establish in amputees, but similar experiences were seen in some of the missing finger RHI participants. A large subsection of these participants could also describe the perceived shape and form of the finger even though they could not see it and additional sensations such as numbness, tingling, and other paresthesias were reported by around 25% of people. The missing finger illusion elicits a range of sensory phenomena which are associated with PLPh and may, therefore, provide a useful method for investigating the underlying mechanisms of some phantom limb experience.

However not all aspects of PLPh were replicated by this illusion. Firstly, the variation in position of the phantom and movement of the phantom, both of which occur in some real phantom limb cases, could not be elicited by the illusion due to the static position of the rubber hand. Secondly, noticeably absent from the range of experiences reported by our participants is any feeling of physical pain due to the missing finger illusion. This may be unsurprising given that none of our participants had actually undergone a traumatic amputation of their ring finger. However, it may also suggest that different mechanisms underlie non-painful and painful sensory phenomena. It is now well documented that phantom limb pain after upper limb amputation is associated with cortical re-organization of the primary somatosensory and motor cortices of the brain (Lotze et al., 2001; MacIver et al., 2008). Functional brain imaging studies show that activation of the lip/face area extends beyond its cortical boundaries to incorporate cortex normally devoted to processing information from the hand/arm. Furthermore, the intensity of the pain is positively correlated with the extent of re-organization, which can be reversed using an intervention based on mental imagery where amputees imagine moving the phantom limb (MacIver et al., 2008). At present it is unclear what causes such extensive re-organization to occur, although there are several theories (see Subedi and Grossberg, 2011, for a recent review). One theory suggests that it is the lack of afferent input to primary sensory cortex, which results in re-organization as the brain utilizes redundant cortex. This highly influential theory has provided the rationale for treatments for phantom limb pain, such as the mirror box (Ramachandran and Rogers-Ramachandran, 1996), which aim to restore afferent sensory input and provide motor feedback, although mirror therapy in general has had mixed results in randomized controlled trials (Brodie et al., 2007; for a discussion of this see Moseley et al., 2008).

The phantom pain and sensation may have its onset immediately or years after the amputation. There are reports of two peak periods of onset, the first within a month and the second a year after amputation (Schley et al., 2008). If the pain associated with phantom limbs is due to cortical re-organization, we would not expect to see this in the short time period over which we tested participants. Non-painful phenomena widely reported in the phantom limb literature and elicited in our study likely precede the development of pain, which takes longer to establish. One possibility is that non-painful phenomena are dependent upon altered multisensory correlations following amputation, which occur over shorter time periods. Cortical re-organization may then occur as a response to these altered multisensory inputs as the brain attempts to re-associate or adapt cortical areas to the objective form of the body. This proposed mechanism for the development of phantom limb pain is based on the idea of the “body schema” (Head and Holmes, 1911). The body schema can be thought of as a template of the entire body in the brain. Any change to the body, such as an amputation, results in the perception of a phantom limb. More recently, Melzack (1989) has proposed that the body schema is formed through a “neuromatrix,” which is a network of neurons that integrate inputs from the somatosensory, limbic, and visual and thalamocortical regions of the brain and a “neurosignature,” which is the patterns of brain activity that are constantly updated based on the conscious awareness of the bodily self (see also Iannetti and Mouraux, 2010). Together, they form output patterns, which can determine pain and meaningful bodily experience, such that deprivation of sensory input from the limbs leads to disruption of the neuromatrix, an abnormal neurosignature, and the development of pain. In addition to sensory-motor cortex, the parietal and frontal lobes have also been shown to be involved in both the normal and abnormal multisensory representation of the limb (Lloyd et al., 2002; Ehrsson et al., 2004) and may underlie phantom sensations including pain (McCabe et al., 2005). Future studies using functional brain imaging should help to determine whether cortical re-organization occurs due to an absence of sensory input or due to an altered pattern of multisensory integration and whether this correlates with subjective pain.

It may appear that, in addition to the absence of pain, another difference between the missing finger illusion and phantom limbs is that the illusion is clearly elicited by external stimulation provided by the experimenter’s mimed stroking of the space where the missing finger would be whereas phantom limbs seem to be the result of internal processes and representations rather than external stimulation. We believe, however, that the current findings point to the possibility that phantom limbs are not the result of purely internal processes but that they too, like the missing finger, are influenced by sensory information coming from the external environment. There are many anecdotal reports suggesting that amputees can feel alterations the phantom experience as a result of interaction with the environment. For example, a phantom limb may recede or telescope to avoid obstacles (Giummarra et al., 2010), textures can be felt through the phantom (Björkman et al., 2010; Weeks et al., 2010). In addition, treatments which are seen to “stimulate” the phantom have been reported to be successful (Huang et al., 2009). In these instances the experience of the phantom limb is felt to change and the amputee attributes it to some physical object seen near to the body. When a sample of amputees who reported PLPh were surveyed about the perceived causes of their phantom experiences, the weather was selected by 47% and the next most commonly selected cause was stress which was selected by 8% (Sherman et al., 1984). These examples demonstrate that amputees sometimes attribute external causes to changes to their PLPh. There is currently no explanation of how this could occur. During normal embodiment, visual cues do not have to be seen on the body to elicit a change in somatic experience. Just seeing a threatening object near the body elicits fear and a physiological stress response and activates parietal brain regions encoding peripersonal space (Lloyd et al., 2006). This ability has been related to the existence of peripersonal space, a region around the body where visual cues are processed as relevant for the body either in terms of reaching, avoidance, or threat detection. Peripersonal space is demonstrated by the existence of cross modal interactions between vision and touch. PLPh may occur due to the same cross modal interactions that support normal embodiment but in a different situation than has ever been experienced before, i.e., when part of the body is missing.

The body changes continuously as we grow up and grow old and the body schema must also change to accommodate these changes. Many experiments demonstrate that changes to the experienced form of the body occur by resolving discrepancies between sensory information. In the extending nose procedure a participant can feel their nose becoming longer when reaching forward and touching another person’s nose whilst a third person touches the participant’s nose (Ramachandran and Blakeslee, 1998). Vibrating a muscle tendon can also lead to the illusory perception that a limb is becoming longer (Lackner, 1988). In these examples the body percept changes because sensory experiences that reference limb position are incongruent. The same perceptual adaptation most likely underlies the changes in size and shape reported during the missing finger illusion. Participants also report changes in the size of the hand during the standard RHI but the perceptual changes are limited to the visual form of the seen hand, which places constraints on the way that embodiment can be manipulated. For example, during embodiment illusions a participant feels their finger or arm stretch when they see it stretch, but the illusion of ownership is lost if part of the body is completely detached (Newport and Preston, 2010; Preston and Newport, 2012). In the present study, the form of the invisible finger changed very quickly and the same participant could report a range of alterations that were attributed to the seen location of the experimenter’s finger. Touch information, from both a tactile and visual senses, shapes the finger percept but when a hand-like object is embodied it is the properties of the object that are adopted. Conversely in the disappearing hand illusion, when there is no body form to see and visual and tactile inputs are absent, there is an absence of embodiment, such that participants cannot feel or locate that body part (Newport and Gilpin, 2011). This situation corresponds to a common sense view of the sensory input that one would expect to be available to an amputee but, in a phenomenological sense, the experience of a phantom limb is more similar to experiences in the missing finger illusion. Collectively this suggests that residual sensory information in the context of an unseen body form may contribute to phantom experience.

The invisible finger phenomena demonstrate how awareness of peripersonal space cannot be thought of as something completely separate from the sense of embodiment. Indeed, they demonstrate that areas of objectively empty space near the body can themselves become part of the subjective embodiment experience. The idea of reciprocal lines of influence connecting sense of embodiment and perception of nearby space is consistent with evidence suggesting that the representation of space near the body changes following amputation. When comparing distances in the landmark position judgment task (Makin et al., 2010), amputees use the intact side of their body in near, but not far, space judgments, suggesting that they come to neglect the space near the missing hand. Space representation is dependent upon body understanding so when it changes, either through illusions, such as the RHI, or through physical alterations to body form such as amputation, the way that space around the body is represented also changes.

An illustration of the intimate connection between the sense of embodiment and peripersonal space can be found in the work of Moseley et al. (2008). A consequence of the RHI is that the temperature of the participant’s real hand is lowered when ownership is transferred to the rubber hand (Moseley et al., 2008). A fall in limb temperature is also measured in CRPS, where damage to the nerves causes people to experience chronic pain and numbness and tingling sensations in the affected body part. These patients show altered tactile processing such that they prioritize tactile information in the unaffected hand over the affected hand. But this effect is reversed when the hands are crossed over the body and tactile input in the affected hand is now prioritized because it rests in the unaffected side of space (Moseley et al., 2009). This again demonstrates that how we experience our own bodies is not just a matter of what is happening within the body itself, but also is affected by the body’s relationship with surrounding objects and spaces.

In this experiment we have demonstrated that an experience similar to that of the phantom limb can be induced in intact participants using a variation of the RHI. We have also shown, through the use of first-person methods, how this experience reproduces various phenomena associated with phantom limbs, although, significantly, not the pain or discomfort that is sometimes associated with phantom limbs. The way in which the experienced invisible finger is felt to grow in length or to otherwise alter its shape as a function of the way in which the experimenter mimicked the stroking of the missing finger indicates how the sense of embodiment is altered by the body’s relationship with its surroundings. Given the similarities between this illusion and PLPh, it may be the case that phantom limbs, and the way that they alter their size and shape over time, are not only a function of an internal body representation but are influenced by relationships within peripersonal space. This, of course, is a matter requiring much further research, but is nevertheless suggestive of possible influences on the way that phantom limbs change over time.

The mediation of sense of embodiment by perception of the surrounding environment has wide philosophical implications, as it suggests that the boundary between self and world is not something absolute and clear-cut. This is a view that has long been advocated by thinkers in the phenomenological tradition, such as Merleau-Ponty (1958) who noted that foreign objects frequently become part of the subjectively experienced body: “The blind man’s stick has ceased to be an object for him… its point has become an area of sensitivity, extending the scope and active radius of touch” (p. 165). The blind person is, nevertheless, still aware of the stick, just not directly as an object, but indirectly via other objects: “In the exploration of things, the length of the stick does not enter expressly as a middle term: the blind man is rather aware of it through the position of objects than the position of objects through it.” (pp 165–166). I am, in other words, “conscious of my body *via* the world” (p. 94). The experiences generated by the RHI in the present study are consistent with Merleau-Ponty’s phenomenological analysis of embodiment.

## Conclusion

In this study we have been able to create an analog of the phantom limb experience in intact participants by using a variation of the RHI in which one of the fingers was missing from the rubber hand. Analysis of first-person reports not only indicated a sense of presence of the missing finger, but the experience among some participants of a number of more specific sensations, such as tingling, associated with phantom limbs. The missing finger version of the RHI may, therefore, provide a means of investigating aspects of embodiment that are difficult to investigate in phantom limb patients themselves. In addition, the way in which the perceived size and shape of the invisible finger altered in the present study indicates that sense of embodiment depends on incoming sensory information from peripersonal space. This is consistent with previous phenomenological work on embodiment and suggests that aspects of the phantom limb experience itself may depend crucially on perception of surrounding space and interactions with the objects in it.

## Conflict of Interest Statement

The authors declare that the research was conducted in the absence of any commercial or financial relationships that could be construed as a potential conflict of interest.
